# Estimating thoracic kyphosis without information on upper thoracic kyphosis: an observational study on 455 patients examined by EOS imaging

**DOI:** 10.1186/s12891-024-07490-2

**Published:** 2024-05-22

**Authors:** Hasan Ghandhari, Mohammad Javanbakht, Farshad Nikouei, Mohammadreza Shakeri, Luca Cegolon, Mohsen Motalebi

**Affiliations:** 1 Shafa Orthopedic Hospital, Iran University of Medical Sciences, Tehran, Iran; 2 Nephrology and Urology Research Center, Clinical Science Institute, Baqiyatallah University of Medical Sciences, Tehran, Iran; 3 University of Trieste, Department of Medical, Surgical and Health Sciences, Trieste, Italy; 4 University Health Agency Giuliano-Isontina (ASUGI), Occupational Medicine Unit, Trieste, Italy; 5 Department of Orthopaedic Surgery, Faculty of Medicine, Baqiyatallah University of Medical Sciences, Tehran, Iran

**Keywords:** EOS imaging, Thoracic kyphosis, Lumbar lordosis, Estimation

## Abstract

**Background:**

Physiological thoracic kyphosis (TK) allows sagittal balance of human body. Unlike lumbar lordosis (LL), TK has been relatively neglected in the literature. EOS is an imaging technique employing high-sensitivity xenon particles, featured by low-dose exposure combined with high accuracy compared to conventional radiography. The aim of this study was to investigate predictors of TK in patients with phyiological spine morphology using EOS imaging.

**Methods:**

EOS images of 455 patients without spinal anomalies were retrospectively assessed for TK (T1- T12), upper thoracic kyphosis (UTK, T1-T5), lower thoracic kyphosis (LTK, T5-T12), LL (L1-S1) and pelvic incidence (PI). The latter curves were measured by two researchers separately and the average of the two measurements was used for further analysis.

Spearman non-parametric correlation was estimated for age, PI, LL, LTK, UTK and TK. Multiple robust linear regression analysis was employed to estimate TK, controlling for the effect of age, sex, LL and LTK.

**Results:**

The mean age of patients was 28.3 ± 19.2 years and 302 (66.4%) of them were females. The mean TK, UTK and LTK was 45.5° ± 9.3, 16 ± 7.4° and 29.7° ± 8.9, respectively. The mean UTK in people under 40 years of age was 17.0° ± 7.2, whereas for patients 40+ years old it was 13.6° ± 7.4. At univariable analysis TK positively correlated with UTK (p<0.001), LTK (p<0.001) an LL (p<0.001). At multivariable linear regression TK increased with LTK (RC = 0.67; 95%CI: 0.59; 0.75) or LL (RC = 0.12; 95%CI: 0.06; 0.18), whereas it decreased with age (RC = -0.06; 95%CI: -0.09;—0.02).

**Conclusion:**

If EOS technology is available, the above linear regression model could be used to estimate TK based upon information on age, sex, LL and LTK. Alternatively, TK could be estimated by adding to LTK 17.0° ± 7.4 for patients < 40 years of age, or 13.6° ± 7.4 in patients 40 + years old. The evidence from the present study may be used as reference for research purposes and clinical practice, including spine examination of particular occupational categories or athletes.

## Background

At sagittal view, the human spine is composed of four curves: cervical lordosis (CL); thoracic kyphosis (TK); lumbar lordosis (LL); and sacral kyphosis (SK) [1]. The distribution of latter curves follows a natural alignment along the sagittal plane, functional to maintain the health of spine and intervertebral discs, improving the performance of movements of the human body while containing energy consumption [2].

TK is often defined as the angle between the superior end plate of T1 and the inferior end plate of T12 vertebrae. TK typically increases with age (particularly after 40 years), yet it may be influenced by various conditions including Schuermann disease, congenital vertebral anomalies or post-traumatic / inflammatory disorders [3].

The standard approach to measure TK at lateral spinal radiography is the Cobb method, where TK corresponds to the angle between two parallel lines, one tangent to the upper end plate of T1 and another tangent to lower end plate of T12. The physiological range of TK varies between 20 to 50 degrees [4, 5]. Likewise, the upper thoracic kyphosis (UTK, T1-T5) and lower thoracic kyphosis (LTK, T5-T12) can be measured in a similar fashion [6, 7]. The mean angle for UTK (T1-T5) is calculated based on routine radiographs, with variability by several factors including geographical area and different study population. Since the quality of the proximal thoracic portion (C1-S1) of lateral conventional radiographs (X ray) of the spine is often unsuitable to visualize the upper T1 endplate [8], a study on 100 healthy individuals older than 40 years suggested to estimate UTK (T1-T5) by adding an extra 14° ± 8 to the angle of LTK (T5—T12) [9]. In view of the above, the present study aimed to estimate UTK in a group of patients with physiological spine morphology using EOS, an imaging technique employing high-sensitivity xenon particles, reportedly featured by low dose radiation exposure combined with higher accuracy compared to CT scan [10, 11]. To the best of our knowledge, UTK was never estimated by EOS imaging thus far.

## Methods

This retrospective observational study was conducted between July 2020 –July 2021 at Shafa-Yahyaian Hospital (Tehran, Iran).

### EOS imaging

EOS (Paris, France) is an imaging technique employing high-sensitivity xenon particles, featured by low-dose exposure combined with high accuracy compared to conventional radiography. With EOS the patient is standing or sitting and a full body image at time is taken from frontal and sagittal views. Despite delivering a much lower radiation dose to the thoracic and lumbar region, the quality of EOS imaging was reportedly higher than CT scan for all anatomical spinal structures, with the exception of the lumbar spinous process [11]. In 2D images of the skeletal system, the radiation dose used by EOS is up to 8—10 times lower than CT scan. For 3D imaging, the dose required for CT-scan reconstruction has been further reduced by 800–1,000 times, yet maintaining an accuracy as good as CT scan [12].

Further advantages of EOS include the possibility of simultaneous imaging of the spine and lower extremities and a relatively shorter preparation time for the radiological image (15 to 30 min less) [10, 13].

**Study Population (New sub-title)**. All patients with a healthy physiological spine curves or with aspecific lumbar pain consecutively accessing Shafa-Yahyaian Hospital (Tehran, Iran) between July 2020 –July 202 to undergo EOS of the thoracic spine were considered in the present study. Patients were excluded if:

1. < 8 years of age and/or;
2. using brace and/or;
3. affected by:
  Thoracolumbar (T12-L1) kyphosis > 10 degrees or lumbar kyphosis of any level, and/or
  Pelvic obliquity, and/or;
  Any deformity or shortening of lower limbs, and/or;
  Any significant deformity along sagittal or coronal plane of the spine needing brace, and/or
  TK > 60 degrees, thoracic scoliosis > 25 degrees or lumbar scoliosis > 15 degrees, and/or;
  History of any type of spine or lower extremities surgery, and/or;
  Acute or chronic or healed spinal fracture or infection.

**Fig. 1:**
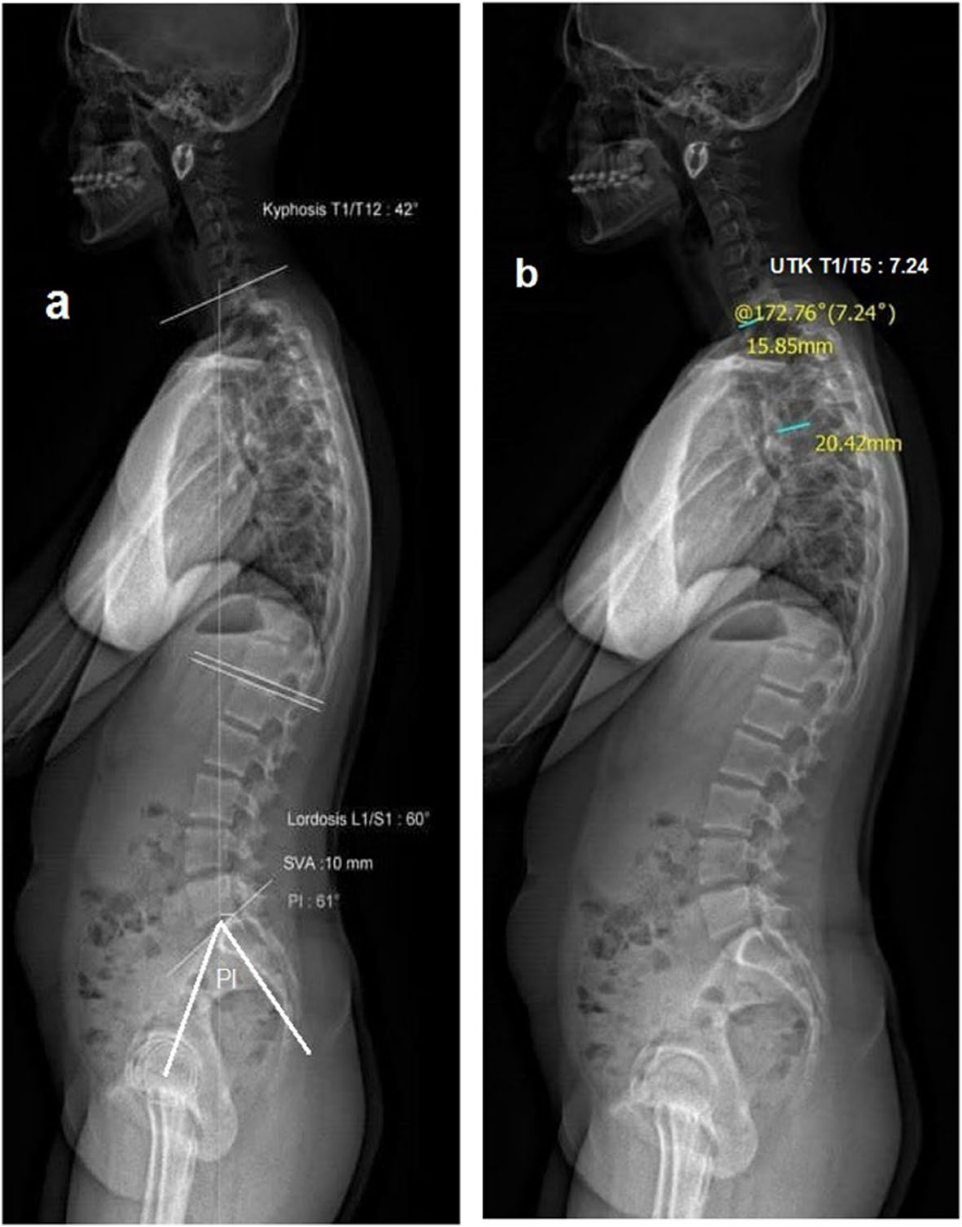
Measurement of sagittal curve angles by EOS imaging. TK= Thoracic kyphosis; UTK= upper Thoracic kyphosis; LL= Lumbar lordosis; PI= Pelvic incidence. **a** TK, LL and PI. **b** UTK

The following measurements were taken from EOS images using sterEOS software and Cobb method (Fig. 1) [14]:

1. TK (paralleled lines with upper T1 and lower T12 endplate), measuring also the angle between them;
2. UTK (angle between upper T1 and lower T5 endplate);
3. LTK (angle between upper T5 and lower T12 endplate);
4. LL (angle between upper L1 and S1 endplate); The average of two patient measurements was considered for further analysis. Pelvic Incidence (PI), which varies between 33° to 85° in the general healthy population, is defined as the angle between the line perpendicular to the sacral endplate at its midpoint and a line connecting this point to the axis of the femoral head [15].

### Statistical analysis

For quantitative variables, median, interquartile range (IQR) and mean ± standard deviation (SD) was calculated, whereas categorical variables were reported as frequencies and percentages. Kolmogorov–Smirnov test was used to assess the normal distribution of quantitative variables.

A multivariable robust linear regression was fitted to investigate factors associated with TK as linear endpoint, reporting adjusted regression coefficients (aRC) with 95% confidence interval (95%CI). Stata 14.0 (Stata Corporation, College Station, Texas, USA) was used for statistical analysis.

## Results

Six-hundred-sixty-three out of 3,482 patients examined by EOS were included in the study.

However, the analysis was eventually restricted to 455 patients whose information on spinal curves was available. In two patients the information on UTK and LTK was not available, due to undetectable anatomical borders of the upper and lower T5 endplates.

Three-hundred-two patients (66.4%) were females. Patients’ age ranged between 5–76 years, with a median of 19 years (IQR; 13–44) and a mean of 28.3 ± 19.2 years. Females were slighly older than males, with a median age of 21 (IQR: 13; 46) vs. 17 (IQR: 13–44) years and a mean age of 29.6 ± 19.4 versus 25.8 ± 17.6 years, respectively (Table 1).

**Table 1:** Distributions of study patients by age and sex

	**Patients**	***N*** ** (%)**	**Range**	**Median (IQR)**	**M ± SD**
**Age** (years)	All	455	8–76	19 (13; 44)	28.7 ± 18.9
	Females	302 (66.4)	8–76	22 (13; 46)	30.0 ± 19.6
	Males	153 (33.6)	8–69	17 (14; 37)	26.3 ± 17.3

*N= *Number, %= percentage, *IQR= *median interquartile range, *M ± SD=* mean ± standard deviation

As can be seen from Table 2, the median TK was 47° (IQR: 40; 53) and the respective mean was 45.5° ± 9.3, while the median UTK was 16° (IQR: 10.9–20.4) and the respective mean was 16° ± 7.4.

**Table 2:** Angle degrees of thoracic kyphosis (*TK*), upper thoracic kyphosis (*UTK*), lower thoracic kyphosis (*LTK*); lumbar lordosis (*LL*) and pelvic incidence (*PI*). Range, median, interquartile range (*IQR*), mean ± standard deviation (*SD*)

**Spinal curves**	**Range**		**Median (IQR)**	**Mean ± SD**
	**Min**	**Max**		
**TK (T1-T12)**	15.0**°**	60.0**°**	47**°** (40–53)	45.5**°** ± 9.3
**UTK (T1-T5)** (Missing: 2)	0.2**°**	41.3**°**	16**°** (10.9–20.4)	16.0**°** ± 7.4
**LTK (T5-T12)** (Missing: 2)	0.7**°**	55.2**°**	30**°** (23.5–35.8)	29.7**°** ± 8.9
**LL ( L1-S1)**	8.0**°**	90.0**°**	57**°** (49–65)	56.6**°** ± 11.5
**PI**	24.0**°**	85.0**°**	48**°** (40–55)	48.3**°** ± 11.2

Table 3 shows the Spearman non-parametric correlation between variables involved. As can be seen:

- TK positively correlated with UTK (*p* < 0.001), LTK (*p* < 0.001) and LL (*p* < 0.001);
- UTK negatively correlated with LTK (*p* < 0.001) and age (p<0.001);
- LTK positively correlated with LL (*p* < 0.001) and age (*p* = 0.020);
- LL positively correlated with PI (p<0.001) (new bullet point) PI positively correlated with age (p<0.001).

**Table 3:**
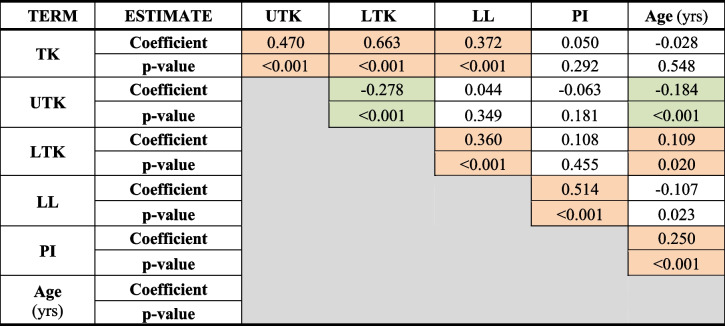
Sperman non parametric correlation. Correlation coefficients with respective *p*-value

Orange highlights mark positive significant correlations. Green highlights mark negative significant correlations

*TK= *Thoracic kyphosis, *UTK=* Upper thoracic kyphosis, *LTK=* Lower thoracic kyphosis, *LL=* Lumbar lordosis, *PI=* Pelvic incidence, *yrs= *years

**Fig. 2:**
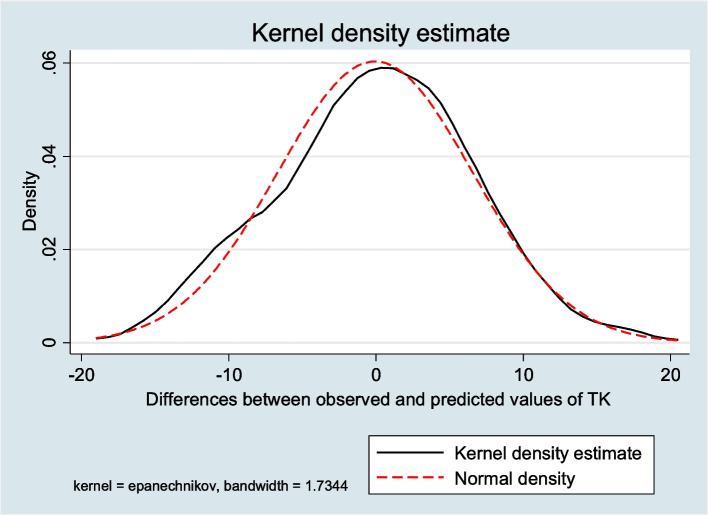
Distribution of differences between observed and predicted measurements of thoracic kyphosis (TK)

Table 4 displays the distribution of TK, UTK and LTK by age and sex. As can be noted, patients aged 40 + years exhibited significantly lower UTK (13.6° ± 7.4 versus 17.0 ± 7.2; *p* < 0.001), yet higher LTK (30.5° ± 9.7 versus 29.4° ± 8.6; *p* < 0.001) and slightly lower TK (44.0° ± 10.0 vs. 46.1 ± 9.0, *p* = 0.031). By contrast, there was no difference in TK by age group in females (*p* = 0.060) or males (*p* = 0.319). Table 5 displays the output of a robust multiple linear regression model including sex, age, LL and UTK. As can be seen, TK increased significantly with LTK (RC = 0.67; 95%CI: 0.59; 0.75) and LL (RC = 0.12; 95%CI: 0.05; 0.18), whereas it diminished with age (RC = -0.05; 95%CI: -0.09; -0.02). The latter multiple linear regression model [TK = 19.03 + (1.23 × sex) – (0.05 × age) + (0.67 × LTK) + (0.12 × LL)], featured by goodness of fit (Fig. 2), could be used to estimate TK if information on UTK is not available (Table 5). Alternatively, TK could be estimated by adding the mean UTK found in the present study (17.0° ± 7.2 for patients aged < 40 years against 13.6° ± 7.4 for patients aged 40 + years) to LTK measured.

**Table 4:** Comparison of thoracic kyphosis (TK), upper thoracic kyphosis (UTK) and lower thoracic kyphosis (LTK) by age and sex. Mean degrees ± standard deviation (M° ± SD); ANOVA *p*-value

**Patients**	**Spinal curve**	**Age**	**M° ± SD**	***P*** **-value**
**All**	**TK**	< 40 years	46.1 ± 9.0	0.031
		40 + years	44.0 ± 10.0	
	**UTK**	< 40 yeras	17.0 ± 7.2	< 0.001
		40 + years	13.6 ± 7.4	
	**LTK**	< 40 years	29.4 ± 8.6	< 0.001
		40 + years	30.5 ± 9.7	
**Females**	**TK**	< 40 years	45.6 ± 9.2	0.060
		40 + years	43.5 ± 10.1	
	**UTK**	< 40 years	16.6 ± 7.0	< 0.001
		40 + years	13.4 ± 7.5	
	**LTK**	< 40 years	29.1 ± 8.4	0.362
		40 + years	30.3 ± 10.0	
**Males**	**TK**	< 40 years	47.1 ± 8.7	0.419
		40 + years	45.1 ± 9.6	
	**UTK**	< 40 years	17.5 ± 7.5	0.013
		40 + years	14.1 ± 7.1	
	**LTK**	< 40 years	29.8 ± 9.0	0.319
		40+ years	31.1 ± 9.0

**Table 5:** Robust multivariable linear regression model estimating thoracic kyphosis (TK)

TERMS		RC (95%CI)	*p*-value
**Constant term**		19.03 (14.90; 23.15)	<0.001
**LTK (linear term)**		0.67 (0.59; 0.75)	<0.001
**LL (linear term)**		0.12 (0.05; 0.18)	<0.001
**Age (years, linear term)**		- 0.05 (-0.09; -0.02)	0.002
**Sex**	**Females**	*reference*	0.077
	**Males**	1.23 (-0.13; 2.60)	

*RC= *Regression coefficients*, 95%CI= *95% confidence interval, *LTK= *Lower thoracic kyphosis, *LL=* Lumbar lordosis*. Multiple linear regression *model fitted onto 453 complete observations

## Discussion

### Key findings

In the present study, the mean TK, UTK and LTK of all patients were 45.5° ± 9.3, 16.0° ± 7.4 and 29.7° ± 8.9 respectively. At multiple regression analysis TK significantly increased with LTK or LL, whereas it decreased with age. If information on UTK is not available, TK could be estimated from LTK, LL and patient age using the above multiple linear regression model. Alternatively, TK could be estimated by adding to LTK the mean UTK calculated in the present study (17.0° ± 7.4 for patients aged < 40 years or 13.6° ± 7.4 in patients 40 + years old).

### Generalizability

Following measurements by Gelb et al. [9] in 1995, if the upper thoracic end plates were not clear at conventional X-ray, TK was estimated by adding 14° ± 8 to LTK, a figure slightly lower than the mean UTK (16.0° ± 7.4) found in the present study. Furthermore, the mean LTK estimated by Gelb et al. was slightly higher (34° ± 11) than that found in the present study (29.7° ± 8.9). The latter discrepancies may be explained by differences in study populations, since all patients in the study by Gelb et al. were > 40 years old and age was inversely correlated with UTK and positively correlated with LTK in the present investigation [9].

For instance, in an Italian study on 160 volunteers aged > 60 years examined by EOS imaging the mean TK was 54.6° ± 13.6, increasing with age (49.4 ± 13.2 at 60–69 years vs. 54.4 ± 13.2 at 70–79 years vs. 63.5 ± 11.3 at 80 + years). Likewise, the mean LTK in the latter study was 47.6° ± 12.6, again increasing with age (44.9 ± 13.7 at 60–69 years vs. 44.9 ± 11 at 70–79 years vs. 46.4 ± 9.1 at 80 + years) [16].

However, a recent systematic analysis on 23 studies reported an average TK of 48.3° ± 6.2, an estimate similar to that found in the present study (45.5**°** ± 9.3) [17]. Likewise, Abrisham et al. reported a mean TK of 43.5° ± 6.4 among 403 patients examined by EOS during 2016–2018 [18].

In the present investigation the mean LL and PI were 56.6° ± 11.5 and 48.3° ± 10.9 respectively. According to the open literature, the mean LL varies widely from 25° ± 11.4 [19] to 64° ± 10 [9], again depending on differences between study populations (in terms of age, race, body mass index, among others), measurement methods and sample size. For instance, whilst Sebaaly et al. [20] reported a mean LL of 59.4° ± 9.9 in 373 caucasian patients aged 18–45 years, Abrisham et al. found a mean LL of 32.4° ± 6.2 [18]. The findings of Abrisham et al. are likely influenced by measurement of LL from upper L1 end plate to lower L5 end plate. L5-S1 disk lordosis has an important role in the restoration of segmental and global LL [21, 22].

 Differently from LL, PI positively correlated with age in the present investigation. However, the latter evidence is still inconclusive – again likely due to differences in study populations [23, 24]. Nevertheless, PI tendd to gradually increase during childhood as a result of bipedal walking, to stabilize after bone maturity [15].

### Strenghts and weaknesses

Despite a generalizability limited by the type of study population and some unmeasured potential confoudners as genetics, race and co-morbidities, the findings of the present study provided some useful reference to estimate TK in patients with physiological spine if information on UTK is not available.

## Conclusions

If UTK is not available, TK could be estimated by the above multiple linear regression model [TK = 19.03 + (1.23 × sex) – (0.05 × age) + (0.67 × LTK) + (0.12 × LL)]. Alternatively, TK could be estimated by adding the mean UTK measurements from the present study (17.0° ± 7.4 for patients < 40 years of age vs. 13.6° ± 7.4 in patients 40 + years old) to the observed LKT.

The evidence from the present study may be used as reference for research purposes and clinical practice, including spine examination of particular occupational categories or athletes. Future studies should investigate sagittal balances by different age groups, conditions and treatment of patients.

## Data Availability

The datasets generated during or analysed during the current study are available from the corresponding author on reasonable request.

## References

[1] Vasiliadis ES, Grivas TB, Kaspiris A (2009). Historical overview of spinal deformities in ancient Greece. Scoliosis.

[2] Abelin-Genevois K (2021). Sagittal balance of the spine. Orthop Traumatol Surg Res.

[3] Katzman WB, Wanek L, Shepherd JA, Sellmeyer DE (2010). Age-related hyperkyphosis: its causes, consequences, and management. J Orthop Sports Phys Therap..

[4] Boseker EH, Moe JH, Winter RB, Koop SE (2000). Determination of “normal” thoracic kyphosis: a roentgenographic study of 121 “normal” children. Journal of Pediatric Orthopaedics.

[5] Fon GT, Pitt MJ, Thies AC (1980). Thoracic kyphosis: range in normal subjects. Am J Roentgenol.

[6] Briggs A, Wrigley T, Tully E, Adams P, Greig A, Bennell K (2007). Radiographic measures of thoracic kyphosis in osteoporosis: Cobb and vertebral centroid angles. Skeletal Radiol.

[7] Greendale G, Nili N, Huang M-H, Seeger L, Karlamangla A (2011). The reliability and validity of three non-radiological measures of thoracic kyphosis and their relations to the standing radiological Cobb angle. Osteoporos Int.

[8] Kuklo TR, Potter BK, Polly DW, O’Brien MF, Schroeder TM, Lenke LG (2005). Reliability analysis for manual adolescent idiopathic scoliosis measurements. Spine.

[9] Gelb DE, Lenke LG, Bridwell KH, Blanke K, McEnery KW (1995). An analysis of sagittal spinal alignment in 100 asymptomatic middle and older aged volunteers. Spine.

[10] Dubousset J, Charpak G, Skalli W, Kalifa G, Lazennec J (2007). EOS stereo-radiography system: whole-body simultaneous anteroposterior and lateral radiographs with very low radiation dose. Rev Chir Orthop Reparatrice Appar Mot.

[11] Deschênes S, Charron G, Beaudoin G, Labelle H, Dubois J, Miron M-C (2010). Diagnostic imaging of spinal deformities: reducing patients radiation dose with a new slot-scanning X-ray imager. Spine.

[12] Glaser DA, Doan J, Newton PO (2012). Comparison of 3-dimensional spinal reconstruction accuracy: biplanar radiographs with EOS versus computed tomography. Spine.

[13] Dubousset J, Charpak G, Dorion I, Skalli W, Lavaste F, Deguise J (2005). A new 2D and 3D imaging approach to musculoskeletal physiology and pathology with low-dose radiation and the standing position: the EOS system. Bull Acad Natl Med..

[14] Yeganeh A, Moghtadaei M, Motalebi M (2018). A challenge on orthopedic sciences: the influence of spinal disease and deformities on total hip arthroplasty: a review on literature. Arch Bone Joint Surg.

[15] Chen H-F, Zhao C-Q (2018). Pelvic incidence variation among individuals: functional influence versus genetic determinism. J Orthop Surg Res.

[16] Bassani T, Galbusera F, Luca A, Lovi A, Gallazzi E, Brayda-Bruno M (2019). Physiological variations in the sagittal spine alignment in an asymptomatic elderly population. The Spine Journal.

[17] Porto AB, Okazaki VH (2018). Thoracic kyphosis and lumbar lordosis assessment by radiography and photogrammetry: a review of normative values and reliability. J Manipulative Physiol Ther.

[18] Abrisham SMJ, Ardekani MRS, Mzarch MAB (2020). Evaluation of the Normal Range of Thoracic Kyphosis and Lumbar Lordosis Angles Using EOS Imaging. Mædica.

[19] Hoseinifar M, Ghiasi F, Akbari A (2007). The relationship between lumbar and thoracic curves with body mass index and low back pain in students of Zahedan University of Medical Sciences. J Med sci.

[20] Sebaaly A, Silvestre C, Rizkallah M, Grobost P, Chevillotte T, Kharrat K (2021). Revisiting thoracic kyphosis: a normative description of the thoracic sagittal curve in an asymptomatic population. Eur Spine J.

[21] Chung N-S, Jeon C-H, Lee H-D, Chung H-W (2021). Factors affecting disc angle restoration in oblique lateral interbody fusion at L5–S1. The Spine Journal.

[22] Hey HWD, Lim JCL, Law GW, Liu GK-P, Wong H-K. Understanding the Pathophysiology of L5-S1 Loss of Lordosis and Retrolisthesis: An EOS Study of Lumbopelvic Movement Between Standing and Slump Sitting Postures. World Neurosurg. 2022;58:e654–61. 10.1016/j.wneu.2021.11.034. 10.1016/j.wneu.2021.11.034 34785359

[23] Bao H, Liabaud B, Varghese J, Lafage R, Diebo BG, Jalai C (2018). Lumbosacral stress and age may contribute to increased pelvic incidence: an analysis of 1625 adults. Eur Spine J.

[24] Zhou S, Xu F, Wang W, Zou D, Sun Z, Li W (2020). Age-based normal sagittal alignment in Chinese asymptomatic adults: establishment of the relationships between pelvic incidence and other parameters. Eur Spine J.

